# Insight into the Desolvation of Quaternary Ammonium Cation with Acetonitrile as a Solvent in Hydroxyl-Flat Pores: A First-Principles Calculation

**DOI:** 10.3390/ma16103858

**Published:** 2023-05-20

**Authors:** Fudong Liu, Shaobin Yang, Xu Zhang, Shuwei Tang, Yingkai Xia

**Affiliations:** 1 College of Mining, Liaoning Technical University, Fuxin 123000, China; m13942496596@163.com (F.L.); xiayingkai200719@126.com (Y.X.); 2 School of Metallurgy and Materials Engineering, Liaoning Institute of Science and Technology, Benxi 117004, China; 3 College of Materials Science and Engineering, Liaoning Technical University, Fuxin 123000, China; zhangxu@lntu.edu.cn (X.Z.); tangsw911@nenu.edu.cn (S.T.)

**Keywords:** desolvation, hydroxyl-flat pore, quaternary ammonium cation, acetonitrile, first-principles calculation

## Abstract

Supercapacitors have a wide range of applications in high-technology fields. The desolvation of organic electrolyte cations affects the capacity size and conductivity of supercapacitors. However, few relevant studies have been published in this field. In this experiment, the adsorption behavior of porous carbon was simulated with first-principles calculations using a graphene bilayer with a layer spacing of 4–10 Å as a hydroxyl-flat pore model. The reaction energies of quaternary ammonium cations, acetonitrile, and quaternary ammonium cationic complexes were calculated in a graphene bilayer with different interlayer spacings, and the desolvation behavior of TEA^+^ and SBP^+^ ions was described. The critical size for the complete desolvation of [TEA(AN)]^+^ was 4.7 Å, and the partial desolvation size ranged from 4.7 to 4.8 Å. The critical size for the complete desolvation of [SBP(AN)]^+^ was 5.2 Å, and the partial desolvation size ranged from 5.2 to 5.5 Å. As the ionic radius of the quaternary ammonium cation decreased, the desolvation size showed a positive trend. A density of states (DOS) analysis of the desolvated quaternary ammonium cations embedded in the hydroxyl-flat pore structure showed that the conductivity of the hydroxyl-flat pore was enhanced after gaining electrons. The results of this paper provide some help in selecting organic electrolytes to improve the capacity and conductivity of supercapacitors.

## 1. Introduction

As a new type of green energy storage component, the supercapacitor has broad application prospects in the fields of vehicle power supply, aerospace, electric energy storage, and so on. It has many desirable properties, including high capacitance, long life cycle, short charging time, high power and energy density, low pollution, and reliable operation [1,2,3,4,5]. Organic electrolytes have become commonplace in the current supercapacitor market because of their higher conductivity, wider electrochemical window, better chemical and thermal stability, and acceptable cost. Organic electrolytes generally use lithium salt quaternary ammonium salt as the electrolyte, and add acetonitrile (AN) and other solvents as needed [6,7]. Graphene is the most commonly used material for electrodes in supercapacitors; it has good electrical, thermal, and mechanical properties and has excellent prospects for application in high-performance energy storage devices, innovative materials, high-performance composite materials, and other fields [8,9]. The phenomenon of metal deposition on the negative electrode and incompatibility with graphene often occur in lithium salt electrolyte capacitor systems [10,11]. Quaternary ammonium electrolytes circumvent the problems that arise with lithium salt electrolytes [12], so that solvent molecules and quaternary ammonium cations are not simultaneously present in the graphene layer. The interlayer spacing of the graphene bilayer can be adjusted to obtain the desired size of the hydroxyl-flat pore [13]. In a related work, two different forms of graphene bilayer, AA and AB stacks, were used to simulate flat pores and to study their desolvation behavior regarding [Li(H₂O)]^+^ [14]. There are few reports on the desolvation behavior of hydroxyl-flat pores to quaternary ammonium salt cationic complexes.

In this study, the desolvation behavior of quaternary ammonium cations (TEA^+^ and SBP^+^) in hydroxyl-flat pores with acetonitrile (AN) as a solvent was investigated using first-principles calculations. Because the hydroxyl-flat pores have different stacking patterns, AA stacking was used to simulate the corresponding form of graphene bilayer hydroxyl-flat pores, and AB stacking was used to simulate the staggered structure of graphene bilayer hydroxyl-flat pores. In this experiment, the size of the corresponding desolvation pore size in solution was determined by calculating the reaction energy during the desolvation of the quaternary ammonium cation (TEA^+^ and SBP^+^). The electronic nature of the interaction between the quaternary ammonium cation and the hydroxyl-flat pore is discussed by calculating the density of states (DOS) of the desolvated quaternary ammonium cation embedded in the hydroxyl-flat pores.

## 2. Calculation Method

The calculations in this experiment were based on the tight-binding (DFTB+) [15,16] software module of density functional theory (DFT) [17,18,19], and the exchange–correlation energy was used for the Perdew–Burke–Ernzerhof (PBE) function under the generalized gradient approximation (GGA) [20,21,22,23]. Due to the large number of atoms in the architecture, the Brillouin zone K-point grid [24] was chosen to be 1 × 1 × 1 [25,26,27], and convergence was verified. An intelligent algorithm was used for optimization of the geometry; the error of the total energy in the system was less than 0.05 kcal/mol, the full stress tensor was reduced to 0.1 GPa, the maximum ion displacement was controlled to within 0.01 Å, and the convergence accuracy of the forces acting on the atoms was 0.5 kcal/mol/Å. A vacuum layer of 20 Å was set in the *Z*-axis direction to avoid the periodicity generated by the vertical interactions.

**Figure 1:**
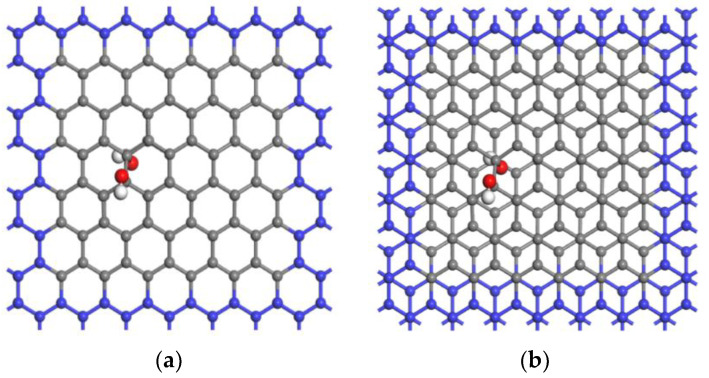
(**a**) Stable structure of AA-stacked hydroxyl-flat pores; (**b**) Stable structure of AB-stacked hydroxyl-flat pores. Hydrogen, carbon, and oxygen atoms are represented by white, gray, and red, respectively.

The graphene bilayer hydroxyl-flat pores can be divided into rigid and flexible pores. However, they change somewhat with the embedding of quaternary ammonium cations during the experiment. We only simulated flexible pores by using edge atoms of a fixed graphene bilayer hydroxyl-flat pore model to improve the pore size and release the elastic pores simulated by atoms in the central region [28]. The two stacked forms (AA and AB) of the graphene bilayer hydroxyl-flat pores studied in this experiment are both composed of periodic hexagonal supercell structures, as shown in Figure 1 (blue atoms on the pore base in Figure 1 are restricted carbon atoms and grey atoms are free carbon atoms), and pores of different sizes (4–10 Å) were simulated by adjusting the interlayer spacing of the graphene bilayer hydroxyl-flat pores.

**Figure 2:**
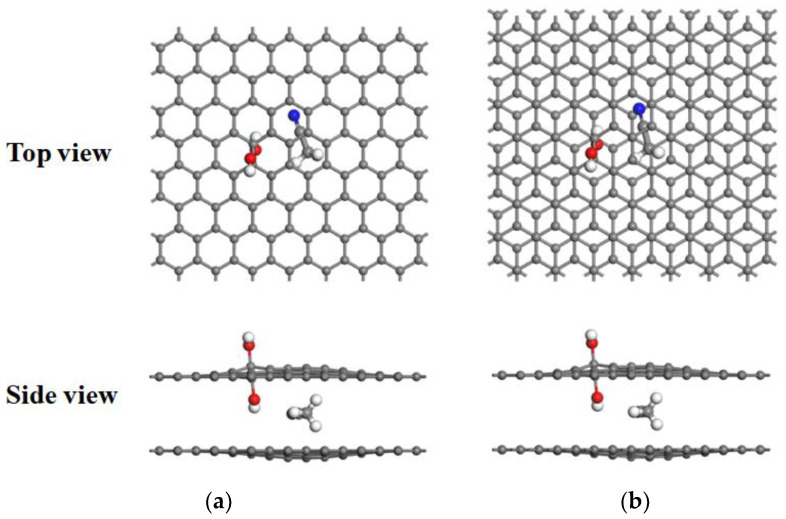
The stable structure model of AN solvent molecular adsorption system. (**a**) AA-stacked hydroxyl-flat pores; (**b**) AB-stacked hydroxyl-flat pores. Hydrogen, carbon, oxygen, and nitrogen atoms are represented by white, gray, red, and blue, respectively.

The addition of a hydroxyl group to the surface of a graphene bilayer flat pore is most stable when the hydroxyl group is adsorbed on a carbon atom. In contrast, the hydrogen atom is most stable when pointed towards the center of the six-circle ring of carbon atoms [29]. The adsorption of hydroxyl groups on the inner side of the flat pores causes one of the three large π-bonds of the planar C-C bond to be broken and the hybrid form to change from sp2 to sp3 and produce unsaturated dangling bonds. Because of this, we chose to adsorb hydroxyl groups on both sides of the graphene flat pores, thus increasing the stability of the graphene bilayer hydroxyl-flat pores [30]. To investigate the intercalation behavior of AN solvent molecules between the layers of graphene bilayer hydroxyl-flat pores with different layer spacing, we investigated their adsorption properties when in different orientations on the graphene bilayer surface and performing orientation energy calculations, as shown in Figure 2. The adsorption position shown in Figure 2 is that with the lowest energy value, i.e., the most stable position for the AN solvent molecules. This is consistent with the viewpoint reported by Leenaerts et al. [31], that adsorption energy is mainly determined by the orientation of the AN solvent molecules.

## 3. Results and Discussion

### 3.1. Reaction Principles

Three reactions may occur during the embedding of quaternary ammonium cations in graphene bilayer flat pores (FP) or hydroxyl-flat pores (HFP): the first is that the quaternary ammonium cations can be stably present in the pores while the AN solvent molecules come off the pores; the second is that the AN solvent molecules can be embedded in the pores while the quaternary ammonium cations are outside the pores; and the third is that the quaternary ammonium cation complexes are embedded in the pores. The three reactions can be represented by Equations (1)–(3), respectively:

A(AN) + FP/HFP → A(FP/HFP) + AN(1)

A(AN) + FP/HFP → AN (FP/HFP) + A(2)

A(AN) + FP/HFP → A(AN) FP/HFP(3)

where A denotes a quaternary ammonium cation (TEA^+^ and SBP^+^); A(AN) denotes a quaternary ammonium cation complex; FP denotes a graphene bilayer flat pore; HFP denotes a graphene bilayer hydroxyl-flat pore; A(FP/HFP) denotes a quaternary ammonium cation compound embedded in a graphene bilayer flat pore or hydroxyl-flat pore; AN (FP/HFP) denotes an AN molecule combination embedded in a graphene bilayer flat pore or hydroxyl-flat pore; A(AN) FP/HFP denotes a quaternary ammonium cation complex in a graphene bilayer flat pore or hydroxyl-flat pore; A(AN) FP/HFP denotes a quaternary ammonium cation complex embedded in a graphene bilayer flat pore or hydroxyl-flat pore; and A(AN) FP/HFP denotes a quaternary ammonium cation complex embedded in a graphene bilayer flat pore or hydroxyl-flat pore. In order to evaluate the possibility of embedding quaternary ammonium cations, solvated quaternary ammonium cations, and solvents in pores of different sizes, the reaction energies were calculated for each of the three reaction cases and defined as *E*_int1_, *E*_int2_, *E*_int3_, *E*_int4_, and *E*_int5_, respectively. They can be expressed by Formulae (4)–(8):

*E*_int1_ = *E*_A(HFP)_*+ E*_AN_ − *E*_A(AN)_ − *E*_HFP_ (4)

*E*_int2_ = *E*_AN(HFP)_*+ E*_A_ − *E*_A(AN)_ − *E*_HFP_ (5)

*E*_int3_ = *E*_A (AN) HFP_ − *E*_A(AN)_ − *E* _HFP_ (6)

*E*_int4_ = *E*_A(FP)_*+ E*_AN_ − *E*_A(AN)_ − *E*_FP_ (7)

*E*_int5_ = *E*_A (AN) FP_ − *E*_A(AN)_ − *E*_FP_ (8)

where *E*_A(AN)_ denotes the energy of the quaternary ammonium cation complex; *E*_HFP_ denotes the energy of the graphene bilayer hydroxyl-flat pores; *E*_FP_ denotes the energy of the graphene bilayer flat pores; *E*_A(HFP)_ denotes the energy of the quaternary ammonium cation compound embedded in the graphene bilayer hydroxyl-flat pores; *E*_A(FP)_ denotes the energy of the quaternary ammonium cation compound embedded in the graphene bilayer flat pores; *E*_AN_ denotes the energy of the AN molecule; *E*_AN(HFP)_ denotes the energy of the AN molecule compound embedded in the graphene bilayer hydroxyl-flat pores; *E*_A (AN) FP_ denotes the energy of quaternary ammonium cation complexes embedded in graphene bilayer flat pores; *E*_A_ denotes the energy of quaternary ammonium cations; *E*_A (AN) HFP_ denotes the energy of quaternary ammonium cation complexes embedded in graphene bilayer hydroxyl-flat pores; and *E*_A (AN) FP_ denotes the energy of quaternary ammonium cation complexes embedded in graphene bilayer flat pores. Smaller values of *E*_int1_, *E*_int2_, *E*_int3_, *E*_int4_, and *E*_int5_ indicate a higher probability of the reaction occurring.

### 3.2. Desolvation of TEA^+^ Complexes

**Figure 3:**
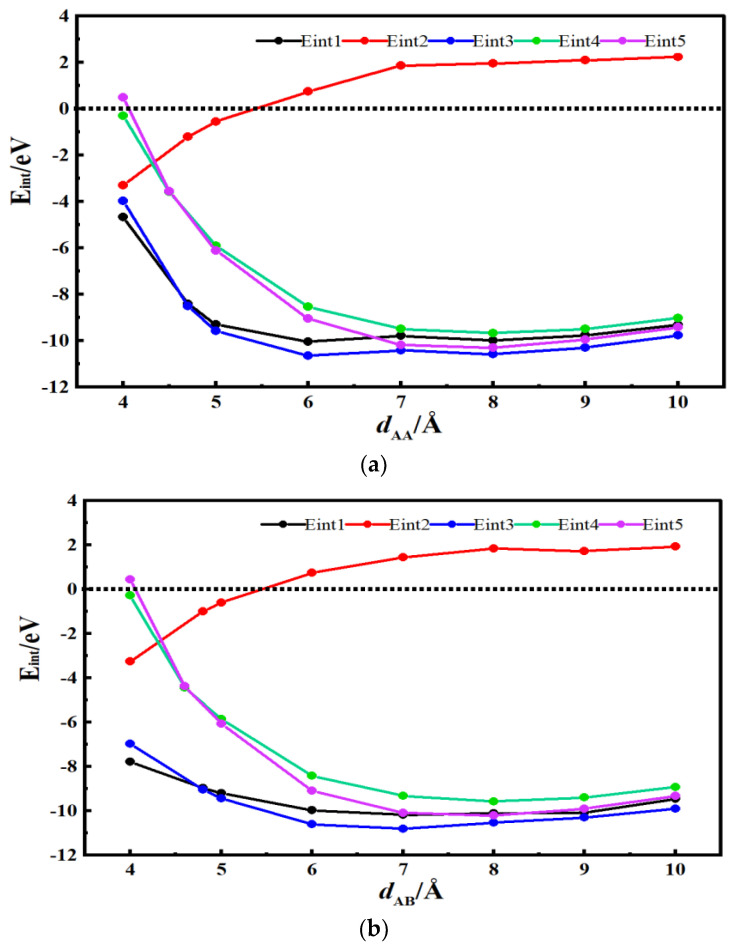
Reaction energy curves for TEA^+^, AN, and [TEA(AN)]^+^ in hydroxyl-flat pores, and TEA^+^ and [TEA(AN)]^+^ in flat pores. (**a**) AA stacking; (**b**) AB stacking.

**Figure 4:**
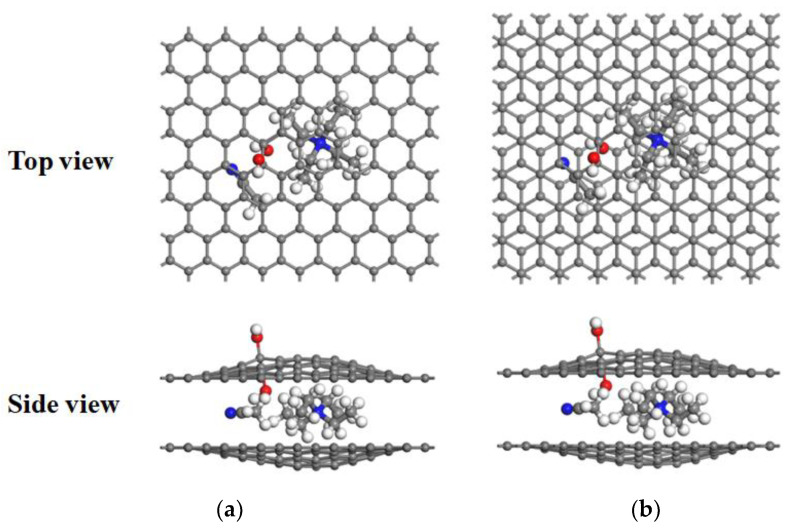
Stable [TEA(AN)]^+^ structure after embedding in hydroxyl-flat pores: (**a**) AA stacking; (**b**) AB stacking. Hydrogen, carbon, oxygen, and nitrogen atoms are shown in white, grey, red, and blue, respectively.

Figure 3a and Figure 4a show the energy curves for the desolvation reactions in AA-stacked graphene bilayer hydroxyl-flat pores. The flat pore model of [TEA(AN)]^+^ was used to obtain the critical size of the desolvation pore size of [TEA(AN)]^+^, and the reaction energies of TEA^+^, AN, [TEA(AN)]^+^ in the hydroxyl-flat pores, and TEA^+^ and [TEA(AN)]^+^ in the flat pores were calculated and expressed as *E*_int1_, *E*_int2_, *E*_int3_, *E*_int4_, and *E*_int5_, respectively. *E*_int2_ < 0 for *d*_AA_ < 5.5 Å indicates that the AN molecule can exist alone in AA-stacked graphene bilayer hydroxyl-flat pores. The energy value of *E*_int1_ (−8.42 eV) compared to *E*_int3_ (−8.50 eV) is similar when the AA-stacked graphene bilayer hydroxyl-flat pores size *d*_AA_ = 4.7 Å, that is, 4.7 Å is the critical point for the desolvation of [TEA(AN)]^+^ in AA-stacked graphene bilayer hydroxyl-flat pores. When the pores are smaller than 4.7 Å and *E*_int1_ < *E*_int3_ < 0, then TEA^+^ is more stable than [TEA(AN)]^+^, and [TEA(AN)]^+^ can undergo complete desolvation. The energy value of *E*_int4_ (−3.58 eV) is similar to that of *E*_int5_ (−3.55 eV) when the AA-stacked graphene bilayer flat pore size *d*_AA_ = 4.5 Å, that is, 4.5 Å is the critical point for the desolvation of [TEA(AN)]^+^ in AA-stacked graphene bilayer flat pores. When these pores are smaller than 4.5 Å and *E*_int4_ < *E*_int5_ < 0, TEA^+^ is more stable than [TEA(AN)]^+^, and [TEA(AN)]^+^ can be completely desolvated.

Figure 3b and Figure 4b show the desolvation reaction energy curves for [TEA(AN)]^+^ in AB-stacked graphene bilayer hydroxyl-flat pore and flat pore models. *E*_int2_ < 0 for *d*_AB_ < 5.4 Å indicates that AN molecules can exist alone in the AB-stacked graphene bilayer hydroxyl-flat pores. The energy value of *E*_int1_ (−8.97 eV) is similar to that of *E*_int3_ (−9.05 eV) at *d*_AB_ = 4.8 Å, that is, 4.8 Å is the critical point for the desolvation of [TEA(AN)]^+^ in the AB-stacked graphene bilayer hydroxyl-flat pores. When these pores are smaller than 4.8 Å and *E*_int1_ < *E*_int3_ < 0, TEA^+^ is more stable than [TEA(AN)]^+^, and [TEA(AN)]^+^ can be completely desolvated. The energy value of *E*_int4_ (−4.43 eV) is similar to that of *E*_int5_ (−4.36 eV) when the AB-stacked graphene bilayer flat pore size *d*_AB_ = 4.6 Å, that is, 4.6 Å is the critical point for the desolvation of [TEA(AN)]^+^ in AB-stacked graphene bilayer flat pores. When these pores are smaller than 4.6 Å and *E*_int4_ < *E*_int5_ < 0, TEA^+^ is more stable than [TEA(AN)]^+^, and [TEA(AN)]^+^ can be completely desolvated. In summary, the two different stacked forms of AA and AB hydroxyl-flat pores and flat pores can exist in solution at the same time. Therefore, the pore size required for the partial desolvation of [TEA(AN)]^+^ is 4.7 Å and 4.7–4.8 Å in bilayer graphene hydroxyl-flat pores, and for the complete desolvation of graphene bilayer flat pores, it is 4.5 Å and 4.5–4.6 Å. TEA^+^ consumes less energy to enter the graphene bilayer hydroxyl-flat pores and is easier to access than the graphene bilayer flat pores. The increase in TEA^+^ in the graphene bilayer hydroxyl-flat pores leads to a rise in supercapacitor capacity. The hydroxyl group increases the adsorption capacity of the flat pores to TEA^+^ and enhances the ability of the flat pores to store TEA^+^.

### 3.3. Desolvation of SBP^+^ Complexes

**Figure 5:**
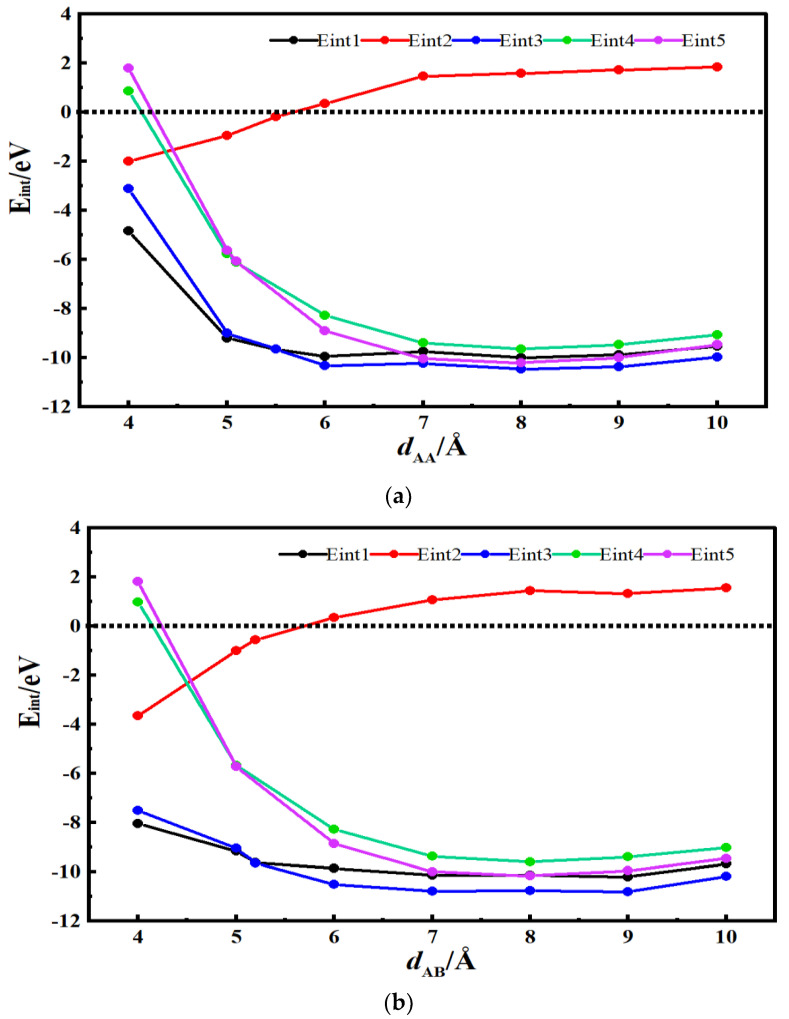
Reaction energy curves of SBP^+^, AN, and [SBP(AN)]^+^ in hydroxyl-flat pores, and SBP^+^ and [SBP(AN)]^+^ in flat pores. (**a**) AA stacking; (**b**) AB stacking.

**Figure 6:**
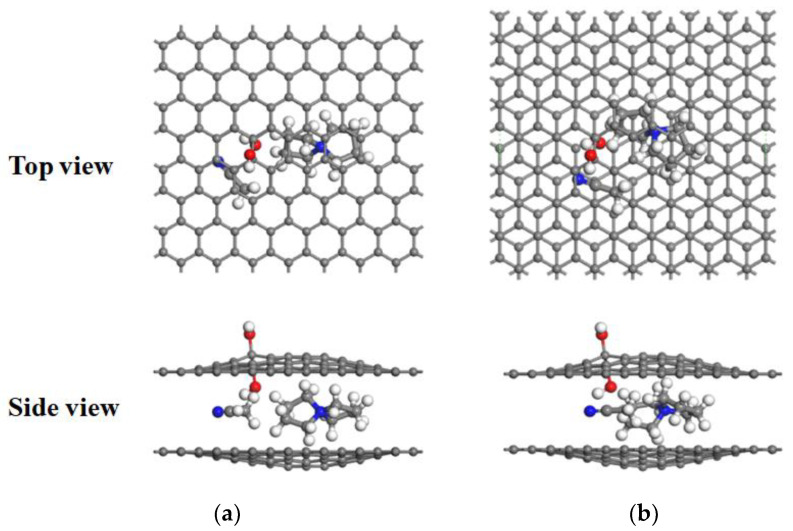
[SBP(AN)]^+^ Stable structure after embedding hydroxyl-flat pores: (**a**) AA stacking; (**b**) AB stacking. Hydrogen, carbon, oxygen, and nitrogen atoms are shown in white, grey, red, and blue, respectively.

Figure 5a and Figure 6a show the desolvation reaction energy curves of the AA-stacked graphene bilayer hydroxyl-flat pore and flat pore model for [SBP(AN)]^+^. To obtain the critical size of the desolvation pore size of [SBP(AN)]^+^, the reaction energies of SBP ^+^, AN, [SBP(AN)]^+^ in the hydroxyl-flat pores, and SBP^+^ and [SBP(AN)]^+^ in the flat pores were calculated and expressed as *E*_int1_, *E*_int2_, *E*_int3_, *E*_int4_, and *E*_int5_, respectively. *E*_int2_ < 0 when *d*_AA_ < 5.7 Å, indicating that the AN molecule can exist alone in the AA-stacked graphene bilayer hydroxyl-flat pores. The energy value of *E*_int1_ (−9.65 eV) is similar to that of *E*_int3_ (−9.64 eV) when the AA-stacked graphene bilayer hydroxyl-flat pore size *d*_AA_ = 5.5 Å, that is, 5.5 Å is the critical point for the desolvation of [SBP(AN)]^+^ in AA-stacked graphene bilayer hydroxyl-flat pores. At AA-stacked graphene bilayer hydroxyl-flat pores smaller than 5.5 Å, *E*_int1_ < *E*_int3_ < 0, meaning SBP^+^ is more stable than [SBP(AN)]^+^ in the pores, so when the pore size is smaller than 5.5 Å, [SBP(AN)]^+^ can be completely desolvated in AA-stacked graphene bilayer hydroxyl-flat pores. The energy value of *E*_int4_ (−6.13 eV) compared to *E*_int5_ (−6.08 eV) is similar when the AA-stacked graphene bilayer flat pore size *d*_AA_ = 5.1 Å, that is, 5.1 Å is the critical point for the desolvation of [SBP(AN)]^+^ in AA-stacked graphene bilayer flat pores. At AA-stacked graphene bilayer flat pores smaller than 5.1 Å and *E*_int4_ < *E*_int5_ < 0, meaning SBP^+^ is more stable than [SBP(AN)]^+^ in the pores, so [SBP(AN)]^+^ can be completely desolvated in AA-stacked graphene bilayer flat pores when the pore size is smaller than 5.1 Å.

Figure 5b and Figure 6b show the desolvation reaction energy curves of AB-stacked graphene bilayer hydroxyl-flat pore and flat pore models for [SBP(AN)]^+^. *E*_int2_ < 0 for *d*_AB_ < 5.8 Å, indicating that AN molecules can exist alone in the AB-stacked graphene bilayer hydroxyl-flat pores. The energy value of *E*_int1_ (−9.63 eV) is similar to that of *E*_int3_ (−9.65 eV) when the AB-stacked graphene bilayer hydroxyl-flat pore size *d*_AB_ = 5.2 Å, that is, 5.2 Å is the critical point for the desolvation of [SBP(AN)]^+^ in these pores. When these pores are smaller than 5.2 Å and *E*_int1_ < *E*_int3_ < 0, then SBP^+^ is more stable than [SBP(AN)]^+^, and [SBP(AN)]^+^ can be completely desolvated. The energy value of *E*_int4_ (−5.68 eV) is similar to that of *E*_int5_ (−5.71 eV) when the pore size of AB-stacked graphene bilayer flat pores *d*_AB_ = 5.0 Å, that is, 5.0 Å is the critical point for the desolvation of [SBP(AN)]^+^ in these pores. When these pores are smaller than 5.0 Å and *E*_int4_ < *E*_int5_ < 0, then SBP^+^ is more stable than [SBP(AN)]^+^, which can be completely desolvated. Therefore, the pore size for the complete desolvation of [SBP(AN)]^+^ in graphene bilayer hydroxyl-flat pores is 5.2 Å, and the partial desolvation pore size is 5.2–5.5 Å. SBP^+^ consumes less energy than the bilayer and so it is easier for it to enter the hydroxyl-flat pores than the flat pores. The increase in the number of SBP^+^ ions allows the supercapacitor capacity to be increased. The hydroxyl group increases the adsorption capacity of the flat pores to SBP^+^ and enhances their capacity to store it.

### 3.4. Analysis of Critical Size for Desolvation of Quaternary Ammonium Cationic Complexes

**Figure 7:**
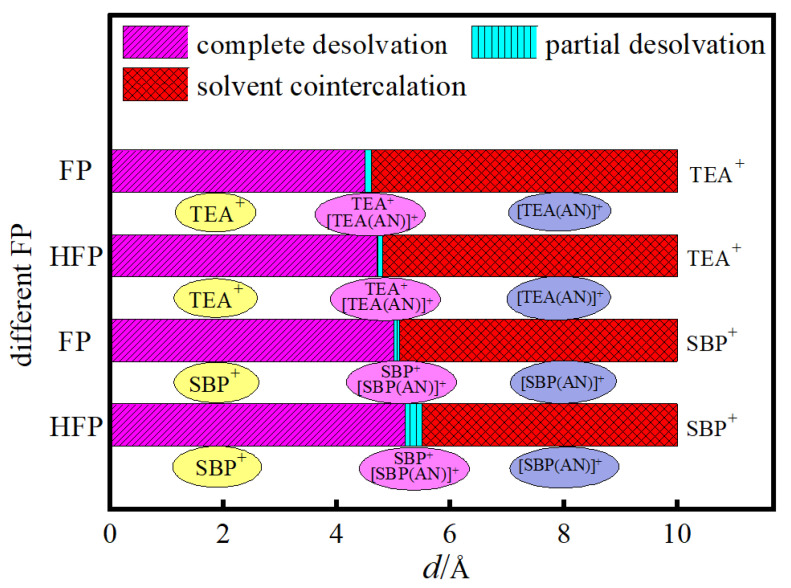
The desolvated sizes of quaternary ammonium cationic complexes in FP and HFP.

The relationship between the desolvation size of the quaternary cationic complexes in graphene bilayer flat pores (FP) and hydroxyl-flat pores (HFP) is presented in Figure 7. Gogotsi et al. [32] demonstrated that microporous (pore size less than 20 Å) materials could significantly increase supercapacitor capacitance and that the desolvation of quaternary ammonium cations can dramatically increase the microporous capacitance. The increase in the desolvation of quaternary ammonium cations inside the micropores significantly increased the supercapacitor’s capacitance. A comparison of the critical size of the desolvation of the same quaternary ammonium cation complex in FP and HFP revealed that the adsorption of hydroxyl groups on the surface of the flat pores increased the capacity of the flat pores to store quaternary ammonium cations, which led to an increase in the capacitance of the supercapacitor. As the radius of the quaternary ammonium cation decreases (3.43 Å for TEA^+^ and 2.09 Å for SBP^+^) [33], the critical desolvation size of TEA^+^ and SBP^+^ increases in the FP and HFP pore models of both AA and AB stacks, and ranges from 4 to 6 Å. Therefore, the critical size for the complete desolvation of [SBP(AN)]^+^ is more significant than that of [TEA(AN)]^+^ in both FP and HFP for quaternary ammonium cations with a larger electric capacity when AN is used as the solvent system. The critical size for the desolvation of the quaternary ammonium cation depends mainly on the ionic radius, which is consistent with the findings of Janes et al. [34].

### 3.5. Density of States (DOS) Analysis

**Figure 8:**
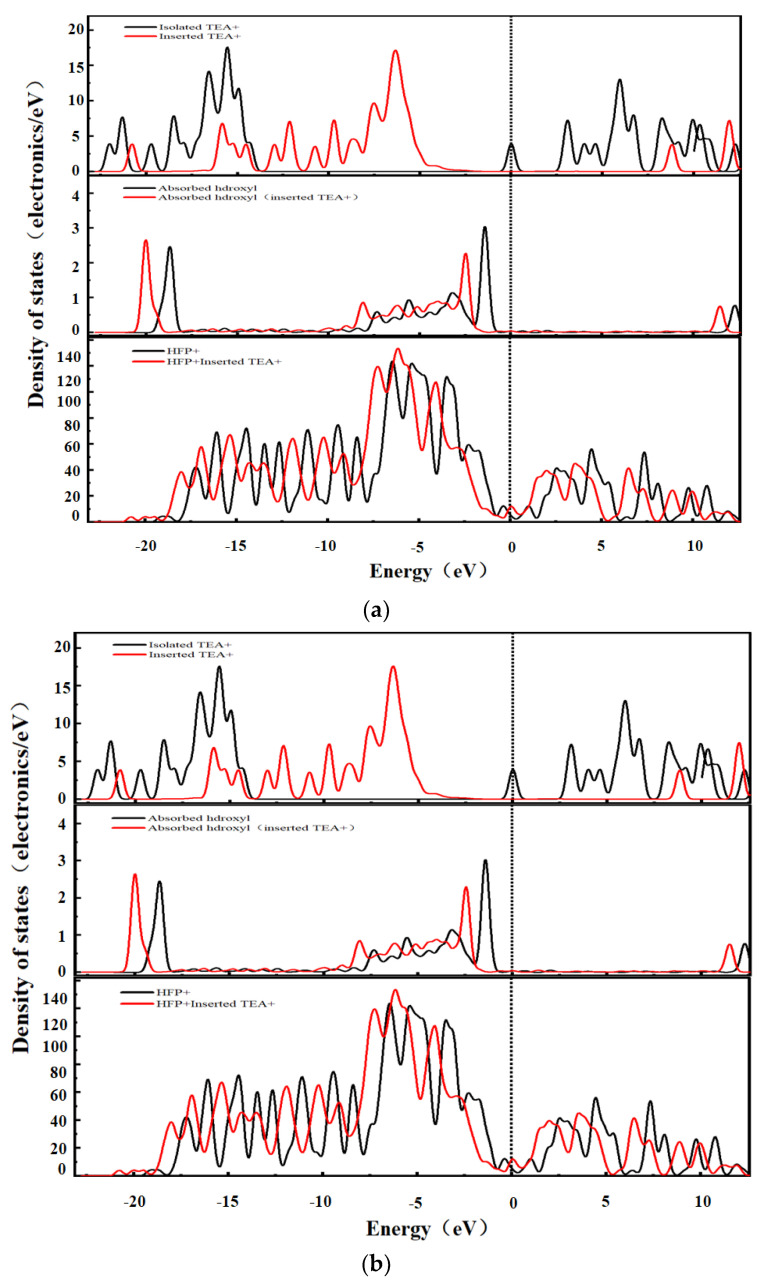
TEA^+^ system density of states: (**a**) AA stacking; (**b**) AB stacking.

To further study the interaction between the desolvated TEA^+^ and the graphene bilayer hydroxyl-flat pores, the density of states (DOS) of the hydroxyl-flat pores of TEA^+^ at the critical size of the desolvation of AA- and AB-stacked graphene bilayer hydroxyl-flat pores and those embedded with TEA^+^ were calculated, as shown in Figure 8a,b. As seen in Figure 8, the AA and AB stacks of the hydroxyl-flat pores do not significantly affect the DOS diagram. The overall position in the DOS diagram of TEA^+^ after embedding in the hydroxyl-flat pores shifts to the right, and the electron orbitals above the Fermi energy level show a non-occupied state, which indicates that TEA^+^ loses a large number of electrons after embedding in the pore. The overall position in the DOS diagram of the hydroxyl group (-OH) shifts to the left after embedding in the pore, indicating that the hydroxyl group (-OH) gains electrons at this time. TEA^+^ does not significantly change the shape of the DOS diagram of the hydroxyl-flat pore after embedding in the AA and AB stacks, but the Fermi energy level shifts upwards, and the conduction band passes through the Fermi surface, indicating that the base of the hydroxyl-flat pore gains a large number of electrons.

**Figure 9:**
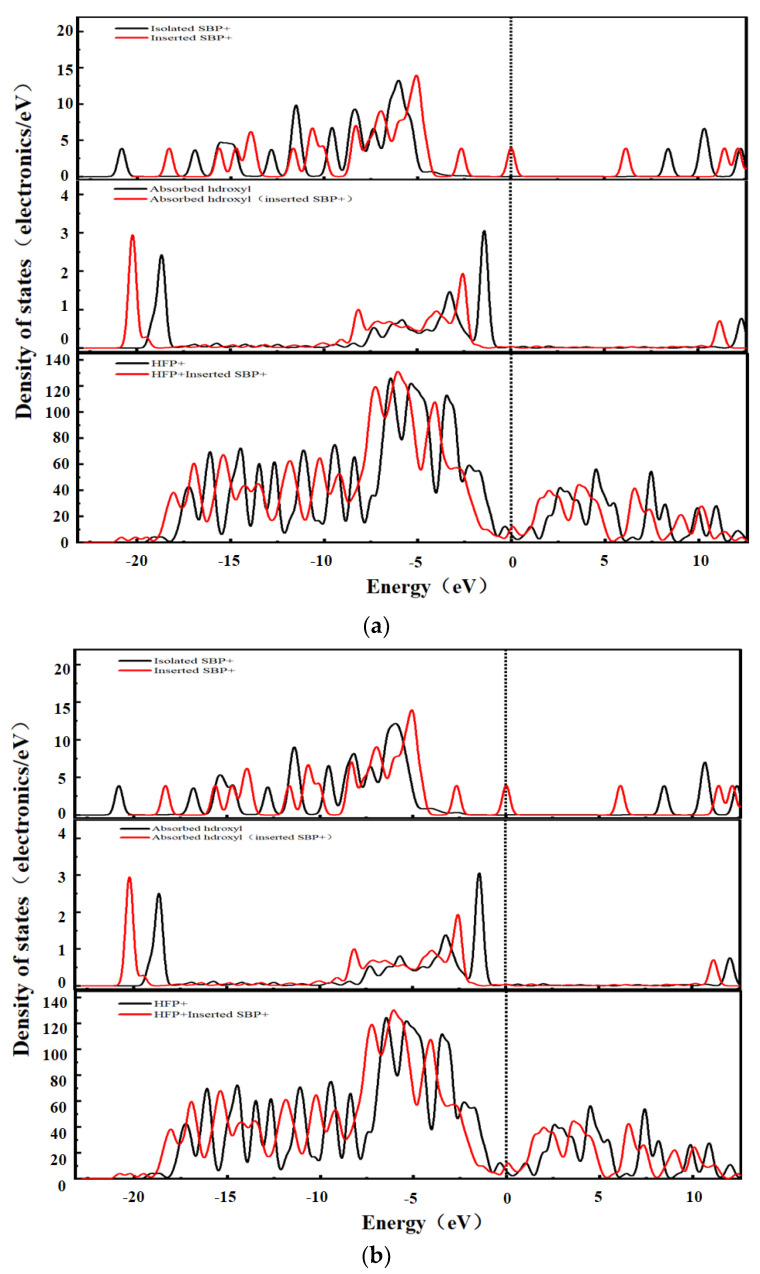
SBP^+^ system density of states: (**a**) AA stacking; (**b**) AB stacking.

The DOS of the hydroxyl-flat pores of SBP^+^ at the critical size for the desolvation of AA and AB stacks is shown in Figure 9a,b. As seen from Figure 9, the AA and AB stacks of the hydroxyl-flat pore do not significantly affect the DOS diagram. The overall position of SBP^+^ in the DOS diagram is shifted to the right after embedding in the hydroxyl-flat pore, and the electron orbitals above the Fermi energy level show a non-occupied state, which indicates that SBP^+^ loses a large number of electrons after embedding in the pore. The overall position of the hydroxyl group (-OH) in the DOS diagram shifted to the left, indicating that it gained electrons at this time. SBP^+^ did not significantly change the shape of the DOS diagram of the hydroxyl-flat pore after embedding in the AA and AB stacks. Regardless, there was a significant increase in the peak on the Fermi surface, and the overall position in the DOS diagram shifted to the left. This indicates that the base surface of the hydroxyl-flat pore embedded with quaternary ammonium cations is metallic, and its conductivity is enhanced after obtaining electrons [35].

## 4. Conclusions

In this experiment, we studied the desolvation behavior of quaternary ammonium cationic complexes in AA- and AB-stacked flat pores and hydroxyl-flat pores with acetonitrile as a solvent using the first-principles calculation method. The complete desolvation flat pore size of [TEA(AN)]^+^ was 4.5 Å, and the partial desolvation pore size ranged from 4.5 to 4.6 Å. The complete desolvation hydroxyl-flat pore size of [TEA(AN)]^+^ was 4.7 Å, and the partial desolvation pore size ranged from 4.7 to 4.8 Å. The complete desolvation flat pore size of [SBP(AN)]^+^ was 5.0 Å, and the partial desolvation pore size ranged from 5.0 to 5.1 Å. The complete desolvation hydroxyl-flat pore size of [SBP(AN)]^+^ was 5.2 Å, and the partial desolvation pore size ranged from 5.2 to 5.5 Å. The adsorption of hydroxyl groups on the surface of the flat pores increased the ability of the flat pores to store quaternary ammonium cations, thus increasing the electric capacity of the supercapacitor. In the acetonitrile solvent system, the critical size of the desolvated quaternary ammonium cation increased with a decreasing radius. The desolvated quaternary ammonium cation lost electrons when embedded in the hydroxyl-flat pores, while the hydroxyl-flat pores exhibited enhanced metallicity and conductivity after gaining electrons.

## Data Availability

Data will be made available on request.
